# Use of nanosystems to improve the anticancer effects of curcumin

**DOI:** 10.3762/bjnano.12.78

**Published:** 2021-09-15

**Authors:** Andrea M Araya-Sibaja, Norma J Salazar-López, Krissia Wilhelm Romero, José R Vega-Baudrit, J Abraham Domínguez-Avila, Carlos A Velázquez Contreras, Ramón E Robles-Zepeda, Mirtha Navarro-Hoyos, Gustavo A González-Aguilar

**Affiliations:** 1 Laboratorio Nacional de Nanotecnología LANOTEC-CeNAT-CONARE, 1174-1200, Pavas, San José, Costa Rica; 2 Universidad Técnica Nacional, 1902-4050, Alajuela, Costa Rica; 3 Laboratorio de Antioxidantes y Alimentos Funcionales, Centro de Investigación en Alimentación y Desarrollo (CIAD), A.C., Hermosillo, Sonora 83304, México; 4 Universidad Autónoma de Baja California, Facultad de Medicina de Mexicali, Lic. en Nutrición, Dr. Humberto Torres Sanginés S/N, Centro Cívico, Mexicali, Baja California 21000, México; 5 Laboratorio BioDESS, Escuela de Química, Universidad de Costa Rica, San Pedro de Montes de Oca 2060, San José, Costa Rica; 6 Laboratorio de Investigación y Tecnología de Polímeros POLIUNA, Escuela de Química, Universidad Nacional de Costa Rica, Heredia 86-3000, Costa Rica; 7 Cátedras CONACYT-Centro de Investigación en Alimentación y Desarrollo A. C., Hermosillo, Sonora 83304, México; 8 Unidad Regional Centro, Departamento de Ciencias Químico-Biológicas y de la Salud, Universidad de Sonora, Hermosillo, Sonora 83000, México

**Keywords:** nanocarrier, nanoformulations, nanosized delivery systems, phenolic compounds

## Abstract

Curcumin (CUR) is a phenolic compound that is safe for human consumption. It exhibits chemopreventive, antiproliferative, antiangiogenic, and antimetastatic effects. However, these benefits can be hampered due to the lipophilic nature, rapid metabolism, low bioavailability, and fast elimination of the molecule. Considering this, the present work reviews the use of CUR-based nanosystems as anticancer agents, including conventional nanosystems (i.e., liposomes, nanoemulsions, nanocrystals, nanosuspensions, polymeric nanoparticles) and nanosystems that respond to external stimuli (i.e., magnetic nanoparticles and photodynamic therapy). Previous studies showed that the effects of CUR were improved when loaded into nanosystems as compared to the free compound, as well as synergist effects when it is co-administrated alongside with other molecules. In order to maximize the beneficial health effects of CUR, critical factors need to be strictly controlled, such as particle size, morphology, and interaction between the encapsulating material and CUR. In addition, there is an area of study to be explored in the development of CUR-based smart materials for nanomedical applications. Imaging-guided drug delivery of CUR-based nanosystems may also directly target specific cells, thereby increasing the therapeutic and chemopreventive efficacy of this versatile compound.

## Introduction

According to the International Agency for Research on Cancer, approximately 9.5 million people worldwide die annually from malignant neoplasms [1]. Deaths are primarily due to cancer of the respiratory system (17%), liver (10%), colon and rectum (8%), breast (8%), and stomach (8%) [2]. The most commonly used treatments involve chemotherapy, surgery, or a combination of both. Chemotherapy is based on the use of molecules such as doxorubicin, paclitaxel, cisplatin, and some proteins and peptides that induce cell death [3]. Conventional or low molecular weight chemotherapeutics remain one of the most common treatment options. Each specific compound will have a particular mechanism of action (alkylating agents, topoisomerase inhibitors), but they all mainly target rapidly dividing cells. However, most tend to be non-specific, thus, they are distributed indiscriminately to all normal and abnormal cells [4], which leads to systemic toxicity that triggers hair loss, loss of appetite, immunosuppression, and inflammation [3]. These side effects can be severe enough to significantly impact the efficacy of a given treatment, while also causing high patient discomfort [5]. Low drug solubility, lack of specificity, high toxicity, poor therapy index, and multidrug resistance are other related drawbacks [6–7]. Orally administered chemotherapy is currently unsuitable due to the low bioavailability of chemotherapeutic agents [8], making intravenous the only viable route of administration, since it avoids bioavailability issues. However, this results in increased toxicity and side effects [3].

Therefore, one of the goals of new and/or improved cancer therapies is for the drug to only target malignant cells in sufficient concentrations and with minimal distribution to other tissues to avoid adverse effects as much as possible. Nanomedicine has recently emerged as an option for cancer treatments and the rationale for its use is based on various improvements seen when a nanosized material and/or drug is administered. It has been successfully used during diagnosis when controlled drug administration is required and for regenerative medicine [9–10], although new applications are constantly being developed. Some of the main advantages of nanosized drug administration include an improved pharmacokinetic profile, higher selectivity towards tumor cells, and increased cellular and organelle internalization, as compared to conventional therapies, potentially increasing therapeutic efficacy and minimizing side effects [4]. Nanosystems can be referred to as nanocarriers, nanoformulations, nanosized-delivery systems, and other similar terms. They have been utilized in the design of cellular and subcellular anticancer therapies which carry the drug and better focus it on the intended target. They are also able to contain molecules that respond to endogenous (tumor microenvironment, including redox potential, pH, enzymes, ions, or biomolecules) and/or exogenous stimuli (light, electromagnetic fields, or ultrasound) [11–12]. Since exogenous stimuli are precisely controlled by the researcher, they can be used to optimize cellular delivery and dose, thus, they will be the main focus of the present work. However, it should be mentioned that those that respond to exogenous stimuli do have some disadvantages, such as the need for additional instrumentation to generate electromagnetic or ultrasonic waves, which may increase their cost and/or complexity.

Curcumin (CUR) or diferuloylmethane is a phenolic compound extracted from turmeric (*Curcuma longa*) rhizomes. It is safe for human consumption [13] and is known for its potentially beneficial health effects [14–15]. Searching for peer-reviewed publications using the term “curcumin” in PubMed, ScienceDirect, and Scopus published during 2010–2020 results in 34.4, 20.4, and 26.6 thousand publications, respectively, of which 74.6%, 62.4%, and 33.4% are cancer-related papers.

The beneficial effects of CUR as an anticancer agent is derived from its chemopreventive [16], antiproliferative [17], antiangiogenic [18] and antimetastatic capabilities [19]. Unfortunately, these benefits can be minimized due to the lipophilic nature, rapid metabolism, low bioavailability, and fast elimination of the molecule [13,20–21]. Thus, research has been focused on incorporating CUR into nanosystems, potentially maintaining or improving its effects on biological systems. The present work reviews the use of CUR-based conventional nanostructured systems used to improve CUR anticancer activity, including liposomes, nanoemulsions, nanocrystals, nanosuspensions, and polymeric nanoparticles, as well as dual effect nanosystems which respond to external stimuli (mainly magnetic nanoparticles and photodynamic therapy), in addition to internal ones. Furthermore, key design factors that could impact the performance of CUR-based nanosystems as a cancer therapy option are also considered. Due to the large body of evidence supporting the bioactivity of CUR, it has been widely considered a suitable molecule to be encapsulated in order to increase and target its delivery. The evidence on this regard was reviewed by Naksuriya et al. [22]. Furthermore, it should be noted that the anticancer bioactivity of CUR is preserved when the parent compound is modified into derivative compounds, such as dimethyl CUR, metal–CUR complexes, tetrahydrocurcumin among others [23].

According to this information, the aim of the present work is to summarize the improvements regarding anticancer effects obtained when CUR is encapsulated using various nanosized systems.

## Review

### Curcumin in cancer therapy

**Figure 1:**
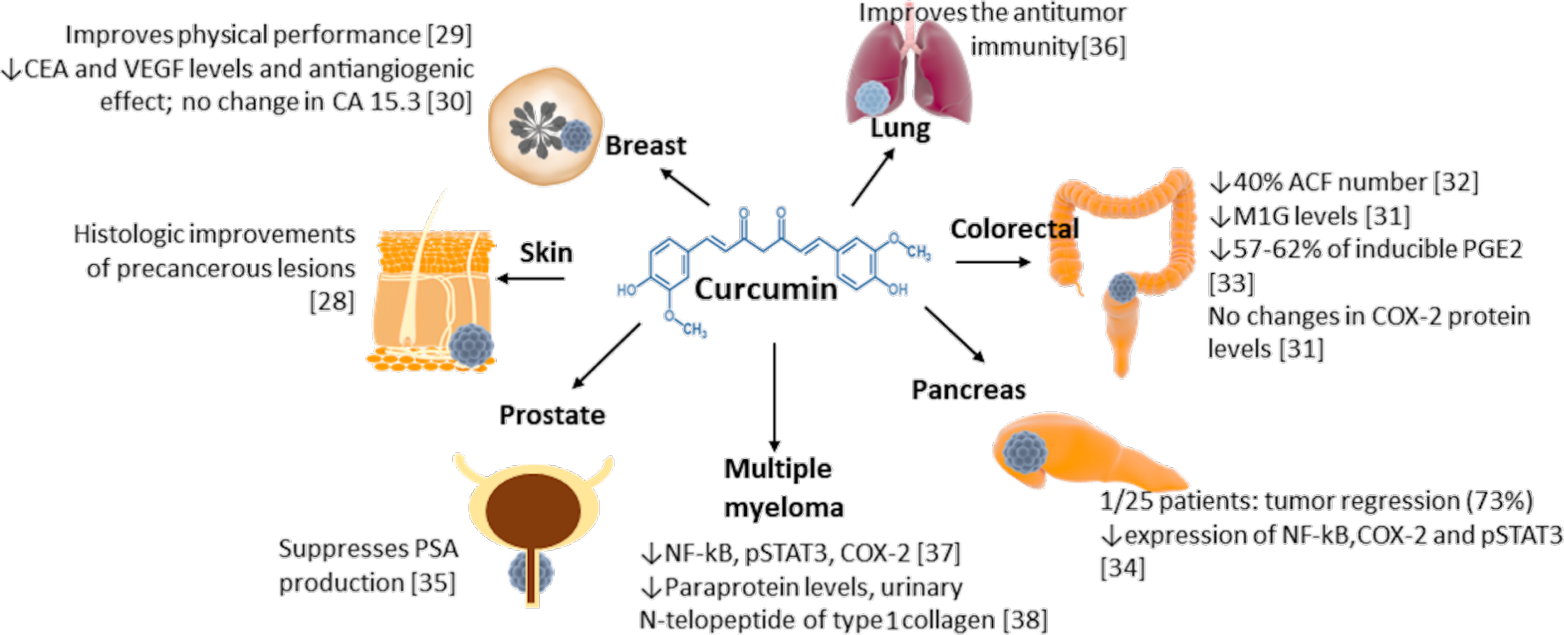
Effects of CUR on cancerous processes observed on clinical trials [28–38]. Carcinoembryonic antigen (CEA); vascular endothelial growth factor (VEGF); cancer antigen 15.3 (CA15.3); DNA adduct 3-(2-deoxy-β-di-*erythro*-pentafuranosyl)pyrimido[1,2-α]purin-10(3H)-one (M1G); enzyme cyclooxygenase-2 (COX-2); colorectal aberrant crypt foci (ACF); prostaglandin E2 (PGE2); 5-hydroxyeicosatetraenoic acid (5-HETE); phosphorylated signal transducer and activator of transcription 3 (pSTAT3); monoclonal gammopathy of undetermined significance (MGUS); nuclear factor kappa B (NF-κB).

Figure 1 summarizes the effects of CUR on cancerous processes, according to information generated from clinical trials. In vitro studies have revealed that the anticancer effects of CUR are mainly due to proapoptotic and antiangiogenic actions, which are regulated by different cell signaling pathways, such as Wnt/β-catenin, PI3K/Akt, JAK/STAT, MAPK, p53, and NF-ĸB [24]. Tumor growth and metastasis are highly dependent on angiogenesis [25], making this process a likely target to prevent, mitigate, or treat some types of cancer. Previous studies have reported the antiangiogenic activity of CUR against cancerous cells [26–27]. In fact, CUR has been shown to hinder proliferation and invasion of non-small-cell lung cancer through the regulation of metastasis-associated protein 1 (MTA1), which is linked to tumor aggressiveness and metastasis, while its modulating effect is related to the suppression of the Wnt/β-catenin pathway. Curcumin also blocks pancreatic cancer metastases through PI3K/Akt/NF-ĸB pathway and induces an antiangiogenic effect in laryngeal squamous cell carcinoma.

The antiangiogenic effects of CUR may be associated to an inhibition of JAK-2 expression, production of pSTAT-3, and decrease in MMP-2 and VEGF, all of which are key vascularization factors. Curcumin also exerts a proapoptotic effect through its interaction with multiple molecular targets, including caspases (3, 9, and 8), Apaf1, PARP, Bax, Cyto-C, PUMA, MCL-1, and Survivin, while also suppressing the expression of β-catenin, p-glycogen synthase kinase-3β (GSK-3β), cyclin D1, c-myc, and the PI3K/Akt/mTOR pathway [24]. In addition to the previous data, it should be stated that coadministration of CUR with another anticancer molecule may also serve to complement or potentiate its effects. The use of a single delivery system will also normalize any differences in the pharmacokinetics of co-encapsulated drugs that are due to different rates of absorption and metabolism, thus, better responses may be achieved [18,39].

### Curcumin nanosystems in cancer therapy

Nanotechnology can improve the effects of conventional chemotherapeutic agents by reducing multidrug resistance, personalizing cancer treatment, decreasing toxicity, and extending product life cycles [7]. Moreover, they can enhance the therapeutic index and pharmacokinetics of several compounds [40]. This is due to their nanoranged size and the possibility of modifications that can make them able to cross biological barriers to reach a specific target organ, cell, or organelle, improving solubility, dissolution rate, and pharmacokinetics [5].

In the case of CUR, the nanosystem size directly influences its biodistribution as demonstrated by Bi et al. [41], who reported differences in the pharmacokinetic profiles when they administered CUR nanosuspension of different sizes (20, 70, or 200 nm). For example, maximum plasma CUR concentration was found 5 min after administration (with concentration of 1250 ng/mL and particle size of 20 nm). In the lungs, spleen, and liver the maximum concentration was observed 10 min after administration (with concentrations and particle sizes of 800 ng/mL-70 nm, 280 ng/mL-200 nm, and 550 ng/mL-200 nm, respectively). In the brain, the maximum CUR concentration was detected 5 min after administration (20 ng/mL with a particle size of 20 nm). This suggests that targeted delivery via an optimized size can lead to an increased organ concentration, making it another important benefit of this nanoscale-based approach. However, it is also possible that the bioactive compound may be instead accumulated in other organs at the same time, not just in the brain. This less specific pharmacokinetic profile could be a disadvantage, since targeting only a particular tissue or organ may not be entirely feasible, thereby causing dosage uncertainties when administering the treatment, which should be taken into account. Thus, there is still room to improve these types of nanosystems, and/or to develop additional ones [42].

### Conventional nanosystems

Conventional nanosystems are particularly attractive for the biopharmaceutics classification system (BCS) class IV drugs, such as CUR, which require increases in their solubilization, pharmacokinetics, and permeation [43]. Curcumin has been targeted against carcinogenic lesions in the colon, skin, cervix, pancreas, prostate, breast, head, and neck [44]. These nanosystems can be lipid-based or polymeric and can contain CUR by itself or in addition to another molecule. General characteristics of these systems and their advantages are described in the following sections. Furthermore, some specific conventional CUR-containing nanosystems that have enhanced therapeutic efficacy are discused.

**Figure 2:**
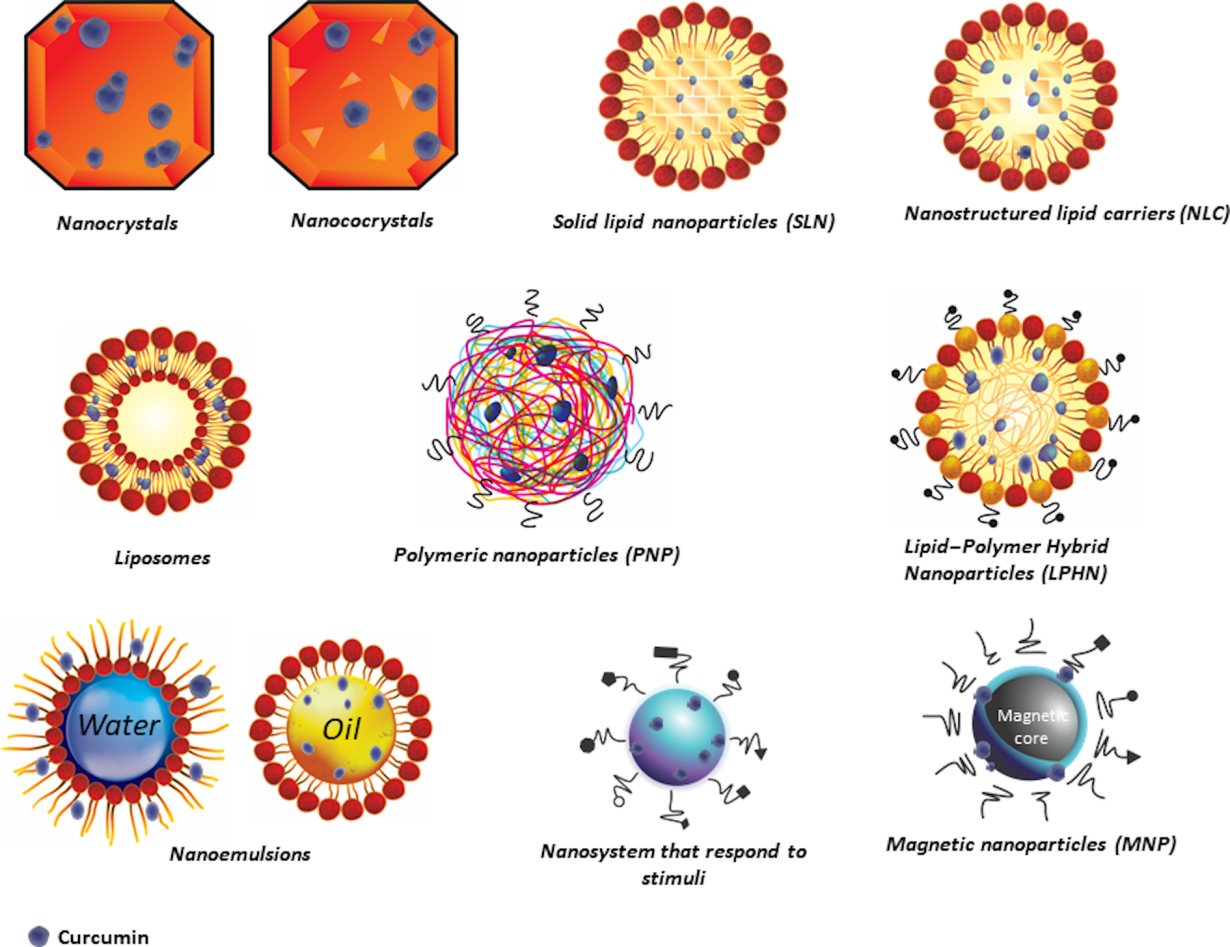
Graphical representation of CUR-based nanosystems. Because of its poor water solubility, CUR will preferentially accumulate in the lipophilic component of the structures, such as the lipid cores or the phospholipids.

**Nanocrystals, nanococrystals, and nanosuspensions.** Nanocrystals are crystalline materials that contain the drug or molecule of interest at the nanoscale, with a size that can range from 10–1000 nm [45]. They can be produced as a powder or as an aqueous suspension containing only the drug [46]. Some authors refer to nanocrystals as indistinguishable from nanosuspensions [47], which may be due to subtle differences between them (Figure 2). Strictly speaking, a nanosuspension is a colloidal dispersion of pure particles of a drug which is stabilized by the use of surfactants [48]. Therefore, a nanosuspension can be prepared by dispersing a nanocrystal or any other powdered material in an outer liquid medium using a stabilizing agent [45]. Furthermore, these nanosystems have shown increased adhesion to surfaces. The advantages of these systems have been evidenced by the significant increase in the in vivo performance of drugs administered in this way [47]. For instance, CUR nanocrystals obtained by solution-enhanced dispersion via supercritical CO_2_ showed increased internalization and apoptotic effects in colorectal cancer cells (HCT116) by promoting cell cycle arrest at the G2/M phase [49]. A CUR nanocrystal has been successfully prepared using melt sonocrystallization, in which its therapeutic potential was evidenced according to in vitro cytotoxicity studies against a human oral cancer cell line (KB). The results showed a growth inhibition (GI_50_) value <10 µg/mL, enhanced inhibition of proliferation, and higher cytotoxicity as compared to free CUR (F-CUR) [50]. Hu et al. [51] formulated a nanocrystal with curcumin and yttrium-stabilized zirconium oxide, which maintained the stability of CUR for 60 days (at 25 °C) and with a dissolution capacity of 98% at 4 h (aqueous solution with 1% SDS, at 37 °C, and 100 rpm). Regarding their pharmacokinetics in vivo (rabbits, 25 mg/kg), it was observed that when the nanocrystal was administered via endotracheal insufflation, the average maximum concentration (*C*_max_) and the plasma area under the curve (AUC) were higher (27.52 mg/L and 37.25 mg/L·h, respectively), as compared to the one administered orally (3.64 mg/L and 11.75 mg/L·h, respectively). The value of *T*_1/2_ was 1.46 h when administered by the pulmonary route and 2.15 h when administered orally, which could be associated with an improved CUR solubility due to the nanosystem. Furthermore, half an hour after pulmonary administration, CUR concentration values higher than 2000 µg/g were observed in the lungs and 6 h after pulmonary administration the concentration was 824 µg/g. Curcumin was also found in other organs, such as the liver (approx. 40 µg/g, 0.5 h), spleen (approx. 30 µg/g, 2 h), kidneys (approx. 19 µg/g, 4 h), heart (approx. 5 µg/g, 4 h), and brain (approx. 5 µg/g, 0.5 h). This shows that CUR nanocrystals administered by the pulmonary route could be a strategy to increase its concentration and permanence in the plasma and in other organs. It should be noted that in a dose-escalation study in human volunteers, in which 10–12 g/day of CUR was administered, only 30–50 ng/mL of curcumin was detected in the serum (between 2 and 4 h after administration), which is associated with its low solubility, instability at a physiological pH, and rapid metabolization [52].

Although nanocrystals make it possible to improve the solubility of CUR, it has been reported that this type of nanosystem can have certain stability problems, which may compromise the ability to ensure a consistent dosage [53].

Nanococrystals, on the other hand, are a combination of two different approaches that include cocrystal preparation and subsequently the nanonization of the obtained cocrystal [54]. The resulting material combines the advantages of cocrystals and nanoscale size to considerably increase solubility, dissolution rate, and bioavailability of the molecules of interest [55]. In addition, as the second molecule in the crystal lattice can also be a bioactive one, their expected effect when used as therapeutic agents can be improved due to their synergic action. This strategy has been used by Ji et al. [56], who modified the surface of CUR nanococrystals with hyaluronic acid to increase its hydrophilicity, which resulted in a better biodistribution. They reported an advantage in the release profile as a function of pH due to a lower CUR release at pH 7.4 (40%) as compared to the acidic pH (pH 5, 80%) which is characteristic of the tumor microenvironment (pH 6.5) and subcellular endosomes (pH 5.0). Regarding its apoptotic effects, a high anticancer activity was found when assayed against a breast cancer cell line (MDA-MB-231), an effect that was accompanied by an enhanced cellular uptake in cells that overexpressed CD44. Besides, they reported complementary evidence in which in vivo studies in female BALB/c mice showed that CUR nanococrystals had better anticancer effects in a 4T1 orthotopic breast cancer model as compared to F-CUR. Ribas et al. [57] reported that supercritical solvents (and no cosolvents) were used to produce their CUR-nicotinamide nanococrystal. The nanosystem showed a higher dissolution rate, in addition to antinociceptive and anti-inflammatory effects.

In contrast to their advantages, cocrystals are not easily obtained. Another option is to formulate a nanoemulsion that contains CUR as the molecule of interest alongside another bioactive entity (i.e., coadministration). Curcumin–piperine nanoemulsion (CUR–PIP) is a great example of this combination approach as a chemotherapy agent against various types of cancer. Piperine has been shown to act as a bioenhancer molecule that improves the solubility CUR while exerting no in vitro antiproliferative effects against HCT116 cells, in either its free or emulsified form [58]. According to the authors, CUR–PIP was more effective, as compared to CUR and CUR emulsion, while PIP and PIP emulsion did not show inhibition. The CUR–PIP nanoemulsion (25 µM-7 µM) inhibited cell proliferation by 50% via cell cycle arrest at the G2/M phase and induced apoptosis. In addition, a CUR nanoemulsion exerted a four-fold increase in caspase-3, in contrast to a six-fold increase exerted by co-administered CUR–PIP. Curcumin is also commonly co-loaded in nanosystems with demethoxy CUR and bis-demethoxy CUR, both of them are related curcuminoids. A nanoemulsion obtained by sonication with Tween 80 and using a *Curcuma longa* extract (curcuminoid concentration 2000 μg/mL, particle size 12.6 nm, encapsulation efficiency 75%, three months after encapsulation and stored at 4 °C) has shown inhibitory effects on A549 (IC_50_ 3.9 µg/mL) and on H460 lung cancer (IC_50_ 2.9 µg/mL) cell lines, according to a cell cycle arrest at G2/M [59]. Morevore, the activities of caspase-3, caspase-8, and caspase-9 had a dose-dependent increase in both cell lines in response to both treatments, which was accompanied by a dose-dependent increase in cytochrome C expression and a dose-dependent decrease in CDK1 expression.

**Solid lipid nanoparticles (SLN).** SLN were first developed ca. 1990 (with patents and papers published a few years after) [60] and are the first generation of lipid nanoparticles (50–1000 nm) [61]. They contain crystallized lipid droplets that are solid at room and body temperatures [62–63], with the drug or the molecule of interest loaded into the solid lipid phase [64]. The advantages of SLN include high drug loading capacity, great stability, good biocompatibility, and improved pharmacokinetics. Their hydrophobicity makes them promising to achieve controlled release and targeted drug delivery to the mononuclear phagocyte system [65]. Effects on the stability of SLN have been reported during storage, mostly due to loss of the solid crystalline structure, which can cause the expulsion of the bioactive compound and, therefore, a rapid initial release [66].

Curcumin-loaded SLN were tested in vitro against MDA-MB-231 cells, which revealed a significantly higher cytotoxicity, cellular uptake, and induced apoptosis, as compared to F-CUR [67]. Curcumin-based SLN have shown increased pharmacokinetics and anticancer activity when the molecule was co-loaded with resveratrol and gelucire and tested in vitro against human colon cancer HCT-116 cells [68].

**Nanostructured lipid carriers (NLC).** Nanostructured lipid carriers are the second generation of drug carriers. They are made up of surfactants and biocompatible lipids that are solid at ambient temperature, which allows them to immobilize drugs and prevent the polymerization of particles within their matrix. Nanostructured lipid carriers have increased drug-loading capacity, as compared to the first generation of lipid-based nanotransporters, due to their liquid-phase lipids [61]. The intestinal permeation of a CUR-loaded NLC has been studied in vitro in Caco-2 cells [69]. The formulation protected the compounds from degradation under basic pH, significantly improved the solubility of CUR, and increased its apparent permeation coefficient. Another study reported a co-loaded CUR–lactoferrin NLC that was prepared as a potential delivery system to cancerous cells through both active and passive targeting [70]. For example, the lactoferrin vector was used for active targeting due to its ability to target tumor cells (mediated by receptors), while the enhanced effect of permeation and retention was considered for passive targeting. Results showed improved in vitro cellular uptake, targeting capability, and cytotoxicity against the human colon cancer cell line HCT116, which was related to early apoptosis after 24 h of incubation.

On the other hand, it has been reported that NLC have a higher loading capacity and greater stability than SLN. This is mainly attributed to the fact that NLC have an imperfect crystalline structure and due to the presence of liquid lipids it prevents the expulsion of the drug [61,66].

**Liposomes.** Liposomes are one of the most well-studied nanosystems. They are self-assembled vesicles with at least one phospholipid bilayer with a diameter size in the range of 50–450 nm when used for medical purposes, and are often used to deliver both hydrophilic and hydrophobic molecules [71–72]. Their main advantages include high biocompatibility, safety, and flexibility (the latter can be modulated by the concentration of cholesterol in the lipid membrane) [40,73–74]. PEGylated long-circulating liposomes with co-encapsulated CUR–doxorubicin inhibited C26 (murine colon carcinoma) cell proliferation, in conjunction with inhibition of angiogenic/inflammatory proteins in an NF-κB-dependent manner [18].

Zhang et al. [75] reported that a combination of nanosized particles and the inclusion of glutathione (which has a thiol group that can initiate thiolysis and release CUR) results in a synergistic effect. This has been previously reported when using glutathione-responsive self-delivery nanocapsules (100 nm), a strategy that increases cellular uptake efficiency, intracellular CUR delivery, and transport to the nucleus, which results in growth inhibition of HeLa (human cervical carcinoma) cells [75]. Similar results have been obtained in glutathione-sensitive PEGylated CUR prodrug nanomicelles [76].

**Polymeric nanoparticles (PNP).** Polymeric nanoparticles are solid colloids with a size of up to 1000 nm; the drug is loaded into the polymeric matrix, preferably using biodegradable polymers [77–78]. If the drug is distributed within a polymeric matrix (i.e., surrounded by the polymer) they are referred to as nanocapsules. Alternatively, if the drug is homogeneously dispersed in the polymeric matrix they are called nanospheres [78–79]. Their main advantages include their reactivity, surface area, stability, and high membrane permeability [80–82]. In particular, their stability tends to increase due to minimal exposure to ambient conditions (thereby delaying oxidation or degradation reactions), while their release may be regulated according to the use of different encapsulating materials [83]. Umerska et al. [84] reported that polymeric nanoparticles formulated with curcumin and an ethyl acrylate copolymer, methyl methacrylate (particle size 231 ± 8 nm, EE% 61.7 ± 4.7, and CE% 56.1 ± 4.3 mg/g) have the advantage of releasing curcumin in aqueous medium (PBS pH 7.4) almost immediately (91% at 60 min), which was associated with the ammonium groups present in the salts of the polymer material. Previous studies have reported that 90% of CUR at pH 7.4 degrades in 30 min [85], so the system developed by Umerska et al. [84] would increase its residence time. Polymeric nanoparticles sensitive to pH exerted an antiangiogenic effect when assayed against human umbilical vein endothelial cells; particles co-delivered doxorubicin–CUR in an amphiphilic poly-β-amino ester copolymer. Their mechanism was apparently related to the VEGF pathway, according to the results of inhibited proliferation, migration, invasion, and tube formation [39]. It was observed that at pH 5.8, more than 90% of CUR and doxorubicin were released, and this amount is significantly higher than that observed at pH 7.4 (59.37% and 66.63%, respectively). This represents an advantage in the systems used to transport drugs to carcinogenic environments because in this type of formulation, it is preferred that the nanoparticle is stable at pH 7.4 (pH of a healthy tissue) and that CUR release is carried out in an acidic pH, which is characteristic of tumor cell endosomes (pH 5.0–5.8) [39]. On the other hand, Bonaccorso et al. [85] compared CUR-loaded polymeric (PNP with PLGA) and lipid nanoparticles (SLN with hydrogenated coco-glycerides and poloxamer 188). They reported greater stability at 135 days (>90% retention), lower average particle size (127.10 ± 11.30 nm), and higher EE% (90.49 ± 1.20%) in CUR-SL, as compared to CUR-PNP (338.20 ± 12.25 nm, EE% 78.04 ± 0.54 >70% CUR). This was associated with the compatibility of the compound with the lipid medium and the presence of the surfactant in the polymer formulation (which has been reported to affect the % EE). In addition, they reported better cellular uptake of SLN, which was associated with smaller sizes and possible internalization by endocytosis (particles with sizes <150 nm can be internalized by clathrin- or caveolin-mediated endocytosis), while particles with sizes from 250 nm to 3 µm can be internalized by macropinocytosis and phagocytosis.

**Lipid–polymer hybrid nanoparticles (LPHN).** Lipid–polymer hybrid nanoparticles have recently emerged as a strategy directed to take advantage of the intrinsic benefits of both liposomes and PNP, as well as to improve drawbacks such as structural disintegration, limited circulation time, and drug leakage. The hybrid materials are constituted by lipophilic and hydrophilic polymers which contribute to increase their systemic mobility and residence time [86]. The advantages of this hybrid system include high encapsulation efficiency, well-defined release kinetics, well-tolerated serum stability, and well-triggered tissue, cellular, and molecular targeting properties [40]. An LPHN was co-loaded with CUR and with docetaxel (82% and 90% average EE, respectively) and assayed in mice bearing PC3 (human prostate cancer) tumor xenografts [87]. The results showed higher cytotoxicity and a synergistic effect, which resulted in tumor growth inhibition without any obvious side effects. Kumar et al. [86] reported CUR-loaded polymer/lipid-based nanoparticles made with the emulsification–evaporation method. They report 53% EE, hemocompatibility in the range of 60–120 µg/mL, and improved apoptotic activity in MCF-7 cells as compared to free CUR [86]. However, both authors only carried out release studies at pH 7.4, so the question of the behavior of LPN at an acid pH remains.

**Nanoemulsions.** Nanoemulsions are composed of an oil phase in a continuous aqueous phase, with average diameters <200 nm [88–89]. The oil and aqueous phases contact each other through a thin interfacial surfactant layer [90]. Some of their advantages include high surface area and high kinetic stability against various types of instabilities. Nanoemulsions have been commercially used to encapsulate CUR and other bioactive agents [91–92]. Their properties allow them to be potential carriers for hydrophobic compounds like CUR, since it can increase its solubility by 1400-fold [90]. When CUR-loaded nanoemulsions were tested, results showed an antiangiogenic effect since there was inhibition of new vessel formation and reduced microvessel density in mice. Moreover, increased CUR solubility and stability and decreased tumor volume were also reported [25]. Table 1 lists the most relevant reports of CUR-loaded nanosystems, while Table 2 summarizes nanosystems in which CUR was co-loaded with another molecule, resulting in an improved chemotherapeutic effect.

**Table 1:** CUR-loaded nanosystems in which significant anticancer effects have been reported.

System	Model (in vitro, except where indicated)	Size (nm), EE (%)	Effects	Ref.

nanocrystals	MCF-7 and A549 cells	153.2 ± 56.4, N.A.	↑ cellular uptake and cytotoxicity	[93]
	in vivo: rabbits and Wistar rats	74, N.A.	↓ systemic toxicity; ↑ concentration in the lungs	[51]
	HeLa cells	100 ± 20, 12–14	no cytotoxicity with 500 μg/mL of CUR	[94]

SLN	MCF-7 cells	152.7 ± 9.9, 96.79 ± 1.81	sustained release; ↓ cell growth; ↑ cellular uptake	[95]
	A549 cells	20–80, 75	↑ drug concentration in target tissue; ↓ xenograft growth	[96]

NLC	U373MG cells	146.8, 90.86	IC_50_ of 9.8 ng/mL; ↑ cellular uptake	[97]
	MG-63 and KHOS cells	98.2, 50	↑ circulation time; ↑ apoptosis	[98]
	KHOS and MCF-7 cells	98.2, 50	↑ effectiveness; ↓ IC_50_	[99]

liposomes	A549 cells	94.66 ± 22.0 N.A.	↑ uptake efficiency	[100]
	B16-F10 and L929 cells	100 ± 6.5, 65.8 ± 4.5	↑ uptake efficiency	[101]
	B16-F10 cells	276.9 ± 14.4, 86.8 ± 6.0	↑ cellular inhibition	[102]
	in vivo: Swiss albino mice	140 ± 60, 72 ± 5	↑ cellular antioxidant enzymes; mitigated mitochondrial destruction and hepatotoxicity; ↓ oxidative damage	[103]
	m-MSCs; in vivo: C57BL66J	377.0 ± 14.6, N.A.	↑ uptake efficiency; ↓ lung metastasis; ↓ tumor growth	[104]
	MDA-MB-231 cells	110, N.A.	↓ growth; ↑ carcinogenic effects	[105]
	in vivo: RG2 (rat glioma) cells	169 ± 4.8, 35 ± 1.2	↑ antitumoral effects; ↓ tumor size; ↑ pharmacokinetics	[106]
	Colon-26 tumor-bearing mice	248 ± 3.98, 65.77 ± 3.17	↓ tumor volume; ↑ cytotoxicity	[107]
	HT29 and HCT116 cells	136, 95	↑ uptake efficiency; ↑ apoptosis	[108]
	B16 (melanoma) cells	202.4 ± 5.2, 97.75 ± 0.5	↑ apoptotic efficiency; ↓ tumor growth	[109]

nanoemulsions	HT29 cells	12.7 ± 0.1, N.A.	↑ apoptosis; ↓ migration of cancer cells	[110]
	non-cancerous human cells (HEK-293T); AGS, HT29, MDAMB-231, and B16-F10 cells in vivo: tumor growth and lung metastasis	200 nm, 95%	cytotoxic on AGS, HT29, MDA-MB-231, and B16F10 cells, safe on HEK-293T cells in vivo: prevents tumor regrowth and spontaneous lung metastasis CUR retention in rats increased in nanoemulsions (15 d), as compared to the compound administered in DMSO (4 d)	[111]

gold nanospheres or nanorods and nanoemulsions in microgels	HEK293T	nanospheres–microgels 220 µm and nanoemulsions–microgels 540 µm	preferential toxicity against cancerous cells	[112]

nanosuspensions	in vivo: hepatocarcinoma mice	186.33 ± 2.73, 67.07	↑ antitumor efficacy; ↓ tumor growth and tumor size	[113]
	PC-3 cells	34.54 ± 2.2, N.A.	↑ apoptosis; ↑ anticancer potential	[114]
	SKOV3 cells	180–222, N.A.	↓ tumor growth; ↑ tumor-bearing mice life span	[115]

EE: encapsulation efficiency; N.A.: not available; SLN: solid lipid nanoparticles; NLC: nanostructured lipid carrier; MCF-7: human breast cancer cells; A549: human lung epithelial carcinoma cells; HeLa: human cervical carcinoma cells; U373MG: brain cancer astrocytoma-glioblastoma cells; MG-63: osteosarcoma cells; KHOS: osteosarcoma cells; B16-F10: murine melanoma cells; L929: murine fibroblast cells; m-MSCs: adipose-derived murine mesenchymal stem cells; C57BL66J: mice with pulmonary melanoma metastasis; MDAMB-231: human breast cancer cells; HT29: human colorectal adenocarcinoma cells; HCT116: human colon carcinoma cells; AGS: human gastric adenocarcinoma; PC-3: prostate cancer cells; SKOV3: human ovarian adenocarcinoma cells.

**Table 2:** CUR co-loaded nanosystems where significant anticancer effects have been reported.

System	Model (in vitro, except where indicated)	Size (nm), and EE (%)	Co-loaded molecule	Effects	Ref.

nanococrystals	in vivo pancreatic cancer	N.A., N.A.	pyrogallol	↓ tumor size	[116]
	U87MG cells	28.79 ± 0.86, 99.2 ± 2.6	“Coformer A”	↑ pharmacokinetics; ↑ distribution in the brain	[117]

NLC	HCT116 and HT29 cells	340.6 ± 33.64, N.A.	ginsenoside	↑ uptake efficiency	[118]
	PC3 cells	122 ± 6, 93 ± 1	genistein	↓ cell viability	[119]

PNP	HT26 cells	204.3 ± 11.0, 35.7 ± 4.1	camptothecin	↑ antitumoral effects; ↑ uptake efficiency	[120]
	K562 cells	248 ± 1.6, 8.6	doxorubicin	↑ cytotoxicity	[121]

nanosuspensions	MCF-7 cells; in vivo Swiss albino mice	80 ± 20, N.A.	docetaxel	↑ cytotoxicity; ↑ uptake efficiency; sensitization of tumor cells; inhibition of p-glycoprotein	[122]

nanoemulsions	PC3 and DU145 cells and murine calvarial osteoblast	137.1 ± 44.6, 98 ± 2.3	etoposide	↑ differentiation; ↑ uptake efficiency	[123]
	SKOV3 cells	144 ± 1.5, 97.4 ± 0.3	paclitaxel	↓ NF-κB activity; modulated P-glycoprotein expression; ↑ cytotoxicity; ↑ apoptosis	[124]

EE: encapsulation efficiency; N.A.: not available; NLC: nanostructured lipid carrier; PNP: polymeric nanoparticles; U87MG: human glioblastoma cells; HCT116: colon cancer cells; HT29: human colorectal adenocarcinoma cells; PC3: prostate cancer cells; HT26: colon cancer cells; K562: myelogenous leukemia cells; MCF-7: breast cancer cells; DU145: prostate cancer cells; SKOV3: human ovarian adenocarcinoma cells.

Guerrero et al. [111] reported the anticancer effects of a CUR-loaded nanoemulsion in an in vivo and in vitro study. Evidence suggests no toxicity against control HEK-293T cells (human embryonic kidney cells), while significant toxicity against AGS, HT29, MDA-MB-231, and B16F10 cells (gastric, colon, breast, and melanoma cells, respectively) was observed when using doses up to 100 μM. In vivo (C57BL/6 mice) data showed that a topical application prevented regrowth and metastasis of excised tumors (melanoma), in contrast to a significant recurrence (70%) in untreated animals. Based on their data, the authors propose that their CUR nanoemulsion can inhibit tumor growth and metastasis, with a particular focus on patients who have undergone surgery to have them excised.

Inostroza et al. [112] report significant effectiveness of a CUR oil-in-water nanoemulsion against gastric cancer cells (AGS). CUR-based treatments were significantly effective antiproliferative agents in concentrations of 12.5–100 μM, while the empty system (without CUR) showed no effect or potential toxicity. Subsequent experiements incorporated the nanoemulsion into an alginate microgel, which the authors argue may be useful as controlled-release delivery mechanisms. This study highlights the versatility of nanoemulsions since their performance can be fine-tuned with the aid of other systems, such as microgels.

### Nanosystems that respond to external stimuli

**Figure 3:**
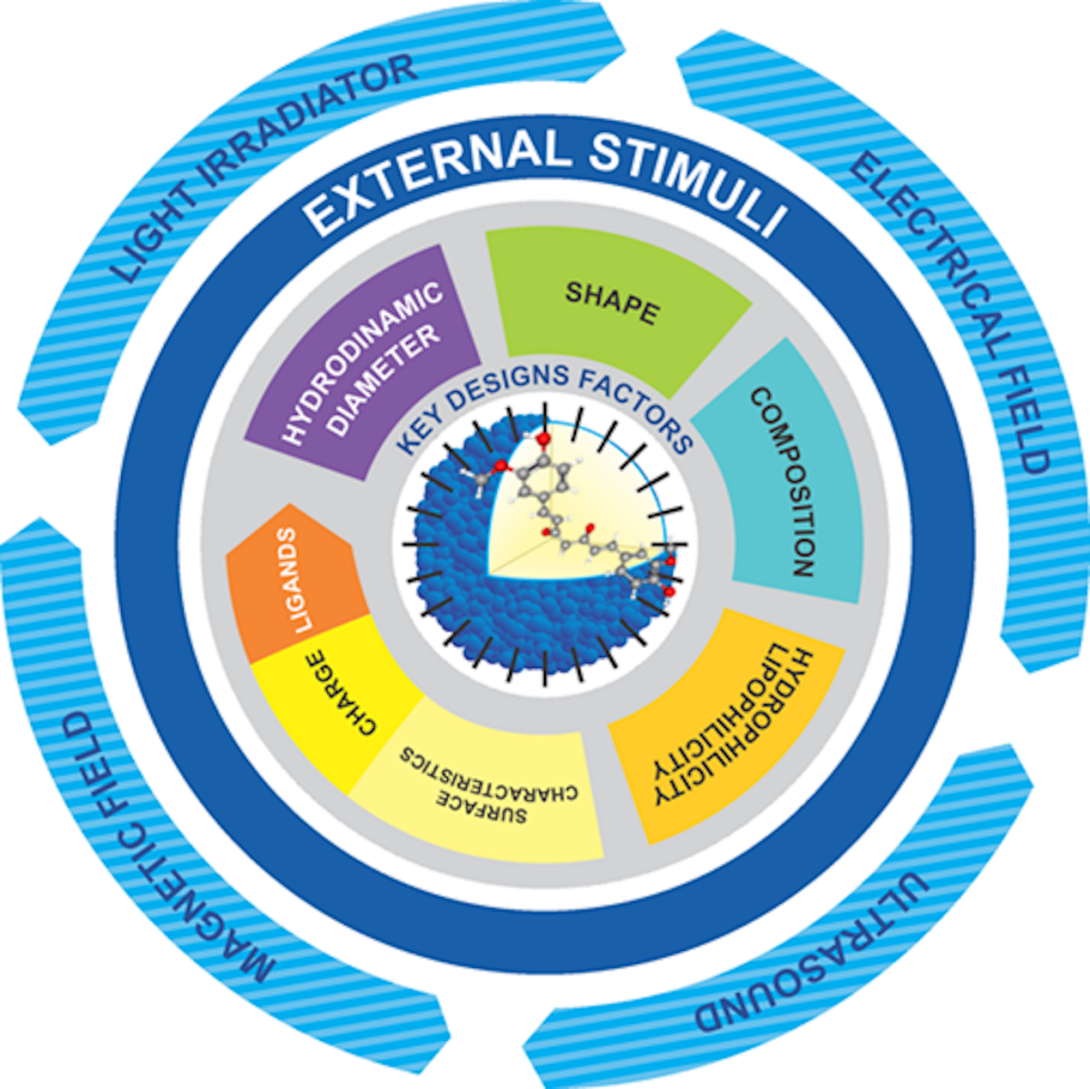
Key factors for the design of nanosystems for targeted cancer therapy.

In addition to their size and other advantages previously discussed, nanosystems can also be prepared such that they release their loaded compound upon receiving an internal and/or external stimulus in order to increase their versatility [12]. Figure 3 summarizes the key factors for the design of nanosystems for targeted cancer therapy. Curcumin-loaded nanosystems have been designed to respond to various external stimuli such as light [125], magnetic fields [126], ultrasound [127] and electric fields [128], or magnetic nanocarriers that respond to changes in pH by increasing the selectivity of the release site [129].

**Magnetic nanoparticles (MNP).** Magnetic nanoparticles contain molecules capable of responding to magnetic fields, which can be magnetically directed to the target tissue where the compound of interest can be released [130–131]. Magnetic nanoparticles can also improve imaging and cause localized hyperthermia (39.5–43 °C), which can be used as a complementary attack strategy against cancer cells [126].

Curcumin-loaded MNP (94.2 µg/mg of nanoparticles) were assayed against MDA-MB-231 cells. They contained iron oxide and β-cyclodextrin and were coated with Pluronic F68 polymer (polyethylene oxide-*co*-polypropylene oxide-*co*-polyethylene oxide) with an average size of 9 nm. Results showed that cells readily internalized MNP by endocytosis, which simultaneously allowed for imaging the affected cells [132]. Other report showed that, using the same breast cancer cells, alginate/chitosan MNP had a 3–6-fold increase in cellular uptake, as compared to F-CUR [133]. This has been shown to increase the antiproliferative effect (IC_50_ 12.4 µM) of CUR, as compared to the free molecule (IC_50_ 17.2 µM) within the assayed range (5–40 µM) [132].

Cui et al. [134] reported the use of CUR-loaded MNP to achieve active targeting in conjunction with transferrin receptor binding peptide T7, which yielded a dual targeting structure that was able to cross the blood–brain barrier, a significant obstacle when treating brain tumors. Similarly, an improved internalization of CUR-loaded MNP was also observed in HPAF-II and Panc-1 human pancreatic cancer cell lines (54.06% and 53.86%, respectively), where a reduced cell proliferation was consequently obtained [135]. In another study, CUR-loaded MNP were significantly more effective in reducing tumor size (71.2%) in xenotransplanted mice (HPAF-II pancreatic cancer cells), as compared to F-CUR (35.9%). This suggests the need to take into account more studies performed on human subjects. Data generated in vitro and in animals should complement the efficacy of a treatment and can be used to determine its mechanisms of action at cellular and molecular scales. However, there is no substitute for human-derived data. Figure 1 summarizes relevant data generated on clinical trials.

Curcumin-loaded MNP had a significantly improved pharmacokinetics in serum (1792.19 ± 644 ng/mL) compared to CUR (766.54 ± 256 ng/mL) [135]. Furthermore, when particles were administered intratumorally (10.5 ± 0.54 nm), nanosystems were detected (mg Fe/g of tissue) in the tumor (1.04) > liver (0.39) > brain (0.14) > spleen (0.06). When administered intraperitoneally, they were detected in the tumor (0.48) and spleen (0.78) [135]. The distribution in other organs may be associated with reduced particle size, improvements in permeability, and tissue retention.

Curcumin-loaded superparamagnetic iron oxide nanoparticles functionalized with sodium dodecyl sulfate (SDS) and coated with chitosan (40–45 nm), were able to induce apoptosis (IC_50_ 30 µg/mL) in HeLa (cervical cancer) cells by damaging the DNA and increasing caspase-3 [136]. Curcumin-loaded, pH-sensitive Janus magnetic mesoporous silica nanoparticles showed a synergistic therapeutic effect in in vivo and in vitro models of liver cancer due to their superior superparamagnetic properties, hyperthermia, high CUR-loading capacity, and sensitivity to the microenvironment of the tumor [137]. Authors argue that the magnetic sensitivity of nanosystems, precise drug delivery, and sustained release support their recommendation to be used as a drug delivery mechanism to induce apoptosis due to hyperthermia (41.85 °C) [136] and higher drug concentration at the target site [138].

**Photodynamic nanosystems.** Photodynamic therapy is based on using light to stimulate photosensitive molecules, which absorb the energy of the photons and interact with oxygen, subsequently producing reactive species that promote cytotoxicity, immunostimulation, and/or regulate tumor vascularity [139]. Previous research has reported the potential use of CUR in an anticancer photodynamic therapy [140–141], in which this combined approach induces mitochondria-dependent apoptosis (70% of cell viability inhibition) in a human head and neck cancer cell line (AMC-HN3), as compared to individually using F-CUR (10% inhibition) or phototherapy (50% inhibition). The apoptosis-promoting effect of the phototherapy–CUR combination is the result of increased nuclear fragmentation, nuclear condensation and reactive oxygen species (ROS) generation, loss of mitochondrial membrane potential, increased cytosolic levels of cytochrome C, and regulation of apoptosis-related proteins (caspase-9, caspase-3, and PARP) [139].

Nitric oxide has been reported to promote resistance to photodynamic therapy in cancer cells, possibly due to its vasodilation effects. It activates MMP-9 and downregulates TIMP-1, allowing CUR to contribute to reduce the resistance to photodynamic therapy by inhibiting nitric oxide in cancer cells [14]. Dev et al. [142] reported that CUR-loaded BSA nanoparticles (6 µM) exposed to blue LED light inhibited the growth of glioblastoma stem cells better than F-CUR, which was attributed to an improved sustained intracellular release of CUR. Curcumin-loaded PLGA [poly(lactic-*co*-glycolic acid)] nanoparticles (hydrodynamic diameter of 200 nm) were induced with LEDs, and showed higher photocytotoxicity against SKOV3 human ovarian adenocarcinoma cells as compared to F-CUR. This was associated with an improvement in cellular internalization of the nanosystem and its greater potential to produce ROS [143]. A similar behavior was observed in cervical carcinoma cell lines treated with nanoemulsion-photostimulated CUR, where cell viability was reduced to <5% and an increase in caspase-3 and caspase-7 activity was apparent [144].

Inostroza et al. [112] reported the promising application of gold nanostructures with CUR-loaded alginate microgels, which showed photothermal properties. High CUR retention and preferential toxicity against cancerous cells was also shown.

The photoactive agent, wavelength, time of exposure, and distance to the light source are important factors that determine the effectiveness of phototherapy against cancer cells [142]. For example, Rahimi-Moghaddam et al. [140] reported a higher cytotoxic effect of a gold–CUR nanosystem used as photothermal therapy against breast cancer cells when exposed to an 808 nm laser (1.5 W/cm^2^), as compared to a 650 nm laser (1.5 W/cm^2^).

The advantages of incorporating CUR into nanostructures used as photodynamic anticancer therapy are diverse. They include making nasal delivery a possibility, thereby minimizing patient discomfort [145], subcellular internalization of the molecule (enhancing sensitization and damage to cancer cells), as well as controlled and constant dosing. Therefore, CUR-loaded nanosystems used as photosensitive therapeutic agents could be an alternative to treat some forms of cancer where similar therapies are already in use (such as cutaneous T-cell lymphoma, basal skin cancer, and esophageal cancer) and to reduce drug resistance. However, further research is necessary to validate its possible beneficial effects in humans. It should also be emphasized that there is significant variation in the metabolism and rate of nanosystems in experimental animals as compared to humans. For example, the type of bond between CUR and the polymeric material is a key factor since it is easier for mice, but not for humans, to break an ester bond than an amide bond [4]. Therefore, the physiological differences between animal models and humans must be considered when studying the effectiveness of nanosystems.

According to previous evidence, the use of nanosystems is highly flexible since it can be tailored for numerous applications with highly specific goals. Polymeric micelles and theranostics (a strategy that combines treatment and diagnostics) are two examples of such applications that are based on customizing the basic nanosystem. Although these are beyond the scope of the present work, their presence should be acknowledged as additional tools, which can be used to potentiate the already high bioactivity of CUR (and other molecules of interest) for anticancer or other health-promoting uses. It is also noteworthy, and a cautionary comment, that CUR has been considered as a candidate molecule for pan-assay interference compounds (PAINS) and invalid metabolic panaceas (IMPS), that is, a compound that interferes with most assay readouts and which appears to exhibit numerous bioactivities [146]. Thus, in vivo experimentation is required to conclusively validate the bulk of in vitro data reported in the literature.

## Conclusion

Curcumin is a bioactive compound whose anticancer effects can be enhanced if administered within a nanosystem. According to independent experimental evidence, a successful formulation must have an optimized particle size and morphology. It can be incorporated into various types of nano-formulations, resulting in increased activity. Co-loading it with other compounds can lead to a synergistic effect or to minimized discrepancies in dosing, since both molecules will be simultaneously delivered. The main data and most conclusive information must be supported by in vivo models (and with human participants in particular), since CUR has been considered a candidate as a PAINS and IMPS compound. Thus, studies and data interpretation must be done with this in mind. Subsequent work in the area must consider the need to prioritize in vivo over in vitro experimentation, although the latter cannot be ruled out since it is an invaluable tool that allows researchers to elucidate mechanisms and mode of action of CUR in nanosystems.
